# Life without Division: Physiology of *Escherichia coli* FtsZ-Deprived Filaments

**DOI:** 10.1128/mBio.01620-16

**Published:** 2016-10-11

**Authors:** Alicia Sánchez-Gorostiaga, Pilar Palacios, Rocío Martínez-Arteaga, Manuel Sánchez, Mercedes Casanova, Miguel Vicente

**Affiliations:** Centro Nacional de Biotecnología, Consejo Superior de Investigaciones Científicas, Madrid, Spain

## Abstract

When deprived of FtsZ, *Escherichia coli* cells (VIP205) grown in liquid form long nonseptated filaments due to their inability to assemble an FtsZ ring and their failure to recruit subsequent divisome components. These filaments fail to produce colonies on solid medium, in which synthesis of FtsZ is induced, upon being diluted by a factor greater than 4. However, once the initial FtsZ levels are recovered in liquid culture, they resume division, and their plating efficiency returns to normal. The potential septation sites generated in the FtsZ-deprived filaments are not annihilated, and once sufficient FtsZ is accumulated, they all become active and divide to produce cells of normal length. FtsZ-deprived cells accumulate defects in their physiology, including an abnormally high number of unsegregated nucleoids that may result from the misplacement of FtsK. Their membrane integrity becomes compromised and the amount of membrane proteins, such as FtsK and ZipA, increases. FtsZ-deprived cells also show an altered expression pattern, namely, transcription of several genes responding to DNA damage increases, whereas transcription of some ribosomal or global transcriptional regulators decreases. We propose that the changes caused by the depletion of FtsZ, besides stopping division, weaken the cell, diminishing its resiliency to minor challenges, such as dilution stress.

## IMPORTANCE

Our results suggest a role for FtsZ, in addition to its already known effect in the constriction of *E. coli*, in protecting the nondividing cells against minor stress. This protection can even be exerted when an inactive FtsZ is produced, but it is lost when the protein is altogether absent. These results have implications in fields like synthetic biology or antimicrobial discovery. The construction of synthetic divisomes in the test tube may need to preserve unsuspected roles, such as this newly found FtsZ property, to guarantee the stability of artificial containers. Whereas the effects on viability caused by inhibiting the activity of FtsZ may be partly overcome by filamentation, the absence of FtsZ is not tolerated by *E. coli*, an observation that may help in the design of effective antimicrobial compounds.

## INTRODUCTION

FtsZ, a cytoplasmic GTPase belonging to the tubulin family, is essential for cell division in *Escherichia coli* (reviewed in references 1 and 2). In liquid medium, cells in which FtsZ is not functional are unable to form septa and grow as long nonseptated filaments (2). When plated at the restrictive temperature, *ftsZ* conditional mutants fail to produce colonies (3). During normal cell division, the FtsZ protein forms a ring at midcell, the FtsZ ring, which initiates the assembly of at least nine additional essential proteins into the divisome (2), the molecular machine that brings about septation.

In *E. coli*, FtsA, a highly conserved member of the actin family, and ZipA, a protein confined to enterobacteria, assemble together with FtsZ, anchoring the resultant proto-ring complex to the cytoplasmic membrane (1). The bifunctional protein FtsK is the next protein to assemble into the division ring, providing a connection between nucleoid segregation and septation. Subsequently, the members of the FtsQ, FtsB, and FtsL complex establish an interaction with the periplasmic space. Assembly is followed by the incorporation of FtsW and FtsI, two proteins related to the synthesis of peptidoglycan. Another essential protein, FtsN, the last protein to assemble at the divisome, is required as a checkpoint for the stable maintenance of the other components of the divisome (4).

In response to the lack of septation, bacteria adopt diverse strategies. In *Bacillus subtilis* and *Staphylococcus aureus* cells, inhibition of division by depleting FtsZ or inactivating it in a thermosensitive mutant or by chemical means leads to the cessation of new DNA synthesis followed by an arrest in cell growth or entry into a stationary-phase-like status that cannot be reverted (5). *Streptococcus pneumoniae* cells, when blocked for division or when entering into the stationary growth phase, suffer autolysis (reviewed in reference 6). In *Streptomyces coelicolor*, *ftsZ* null mutants are viable as membrane compartmentalization and even fragmentation of the mycelium can occur during vegetative growth in the absence of FtsZ (7, 8). Nevertheless, *S. coelicor* ftsZ mutants fail to sporulate because FtsZ is required for septation of the aerial mycelium.

Following cessation of growth and division, the model Gram-negative rod *E. coli* has developed distinct strategies for survival. At the onset of the stationary growth phase, division resulting from the last round of replication occurs, yielding daughter cells of the minimum cell length before stopping altogether (9). In addition alternative sigma factors are induced (10), and the resting population accumulates *surA* mutations in the long term (11). Mutants lacking an active PBP 1B have been described to be effective in proliferating in the absence of FtsZ under conditions of elevated magnesium concentration. These cells, containing a peptidoglycan wall, adopt an aberrant morphology and can sprout viable fragments that grow and divide at rates lower than the normal *E. coli* cells do (12). In this light, we have studied the physiological strategy adopted by growing *E. coli* cells when the amount of FtsZ falls below a critical level.

## RESULTS

### Low levels of the FtsZ protein do not support its assembly into detectable FtsZ rings.

**FIG 1:**
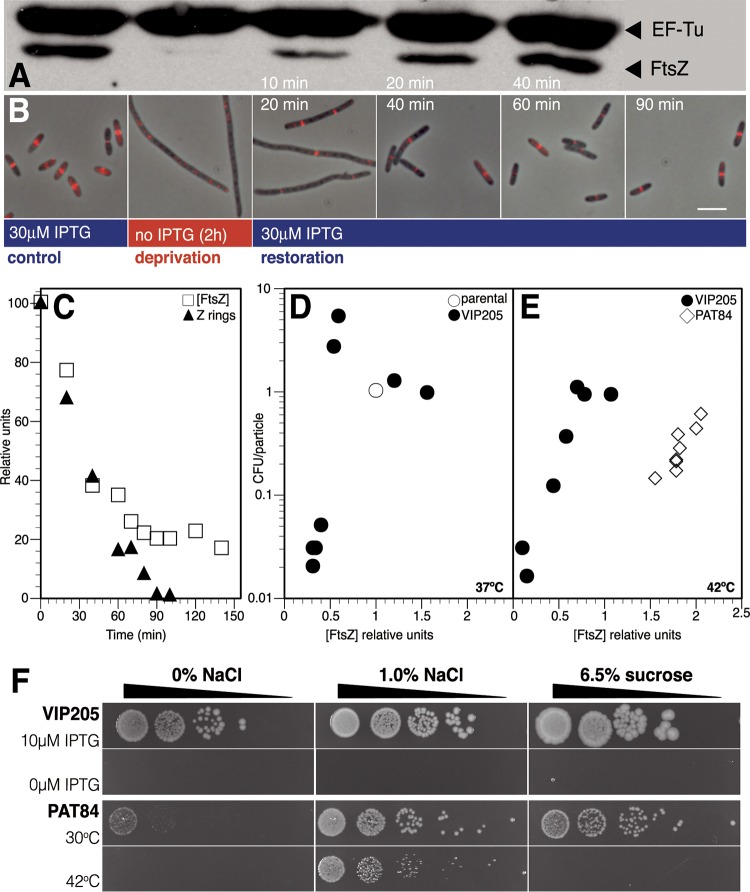
Cell viability, FtsZ levels, and assembly into detectable Z rings after deprivation and restoration of *ftsZ* transcription. Cultures of VIP205 cells growing exponentially in the presence of 30 μM IPTG were transferred at time zero to medium without inducer and sampled at indicated time points during FtsZ deprivation. To study FtsZ restoration, 30 µM IPTG was re-added to cultures after 2 h of deprivation, and samples were taken at the times shown in each panel. (A) Immunoblot showing cellular levels of FtsZ and elongation factor EF-Tu in VIP205 control cells after 120 min of *ftsZ* transcription deprivation and during restoration of *ftsZ* transcription. EF-Tu was used as a control as its concentration remained constant throughout the experiment. (B) Immunolocalization of the FtsZ protein (pseudocolored red) in the same cultures as in panel A. Overlaps of immunofluorescence images with phase-contrast images are shown for the control cells and samples fixed at the indicated times after FtsZ deprivation and subsequent FtsZ restoration. The size bar represents 5 µm. (C) Kinetics of FtsZ protein concentration and FtsZ ring abundance during FtsZ deprivation. The FtsZ concentration was measured and normalized to that of EF-Tu at each time point. The frequency of FtsZ rings in each sample, calculated as the total number of FtsZ rings relative to the total cell length and made relative to the ring frequency at time zero, is shown. Initial control values for protein concentration, relative to EF-Tu, and ring frequency (0.29 rings per µm) were assigned the relative value of 100. (D) VIP205 viability relative to the FtsZ protein levels during *ftsZ* transcription deprivation. The number of CFU per particle as a function of the concentration of FtsZ (compared to its concentration in the parental strain [open circles]) was measured in the samples obtained at times indicated in panel C by plating suitable dilutions on plates containing 10 µM IPTG (see Text S1 in the supplemental material). (E) Plating efficiency relative to the FtsZ protein levels during *ftsZ* transcription deprivation in the strain VIP205 and during upshift to the restrictive temperature of the *ftsZ* functional mutant PAT84. The number of CFU per particle as a function of the concentration of FtsZ (compared to its concentration in each parental strain as in panel C) was measured in the samples obtained at 42°C. (F) Plating efficiency of VIP205 and PAT84 growing at different concentrations of salt and sucrose. Cultures of VIP205 cells growing exponentially in the presence of 30 µM IPTG were transferred to medium with (10 µM) or without (0 µM) inducer at 37°C. Cultures of PAT84 cells growing in LB with 0% NaCl at 30°C were transferred to 30°C or 42°C. Cells at a density of 10^6^ cells/ml were serially diluted 1:10 (ramps show increasing dilution factor), spotted (5 µl each) onto agar plates containing 0 or 1% NaCl or 6.5% sucrose, and grown for 16 h.

To achieve FtsZ deprivation, we removed the inducer to a strain (VIP205 [13]) that contains the *ftsZ^+^* structural gene under control of an IPTG (isopropyl-β-d-thiogalactopyranoside)-inducible *tac* promoter as a single chromosomal copy. To maintain normal morphological parameters at high growth rates, VIP205 cultures need to grow in the presence of 30 µM IPTG (13). This IPTG concentration allows *ftsZ* expression from the P*tac* promoter at levels yielding an FtsZ amount per cell near 140% the levels found in the parental strain (Fig. 1A) (14). To examine the fate of FtsZ rings under conditions of FtsZ deprivation, a culture grown in the presence of 30 µM IPTG was transferred to medium without IPTG, maintaining all other growth conditions unchanged, by making 1/4 dilutions in prewarmed medium as needed to keep optical density values at 600 nm (OD_600_) between 0.2 and 0.3. Samples were removed at intervals to measure the protein levels (Fig. 1A), to immunolocalize FtsZ rings (Fig. 1B), and to determine particle number.

Upon removal of the inducer, FtsZ levels decreased rapidly (Fig. 1A and C), and after 80 min (Fig. 1C) stabilized at a basal level of 20% of the initial value. These FtsZ levels can be attributed to the combined action of a basal expression from the promoter and an increased amount of *zipA* transcript (see Table S1 in the supplemental material) and product (see Fig. S3 in the supplemental material), as ZipA protects FtsZ from degradation by ClpX (15). This residual FtsZ amount failed, nevertheless, to properly localize into detectable rings (Fig. 1B), and FtsZ ring numbers closely followed the kinetics of disappearance of FtsZ (Fig. 1C). For at least six mass doublings, long nonseptated filaments were formed, indicating that cell division was arrested while growth was largely not affected (see Fig. S1 in the supplemental material).

These results suggest that below a threshold concentration, FtsZ fails to assemble as the FtsZ ring, reflecting the highly dynamic nature of the ring (3). Our data indicate that this minimum threshold for the protein to form rings is approximately 20% of the initial level found in VIP205 cells growing in the presence of 30 µM IPTG (Fig. 1B). Under these conditions, VIP205 contains 140% of the amount of FtsZ per cell relative to the parental strain (MC1061) value when growing under similar conditions. From these data, we calculate that FtsZ fails to form rings when its concentration falls below 28% of the amount per cell found in the parental strain.

### Loss of plating efficiency of FtsZ-deprived cells.

To test if filaments formed at small amounts of FtsZ were affected in their physiology (5), we measured the plating efficiency of FtsZ-depleted cells by plating suitable dilutions of the VIP205 culture growing without inducer for a total of eight mass doublings onto plates containing 30 µM IPTG. When the levels of FtsZ were below 50%, the amount present in the wild-type strain, cell viability dropped below 10%, and when FtsZ was present at 20%, it further decreased to 1% (Fig. 1D). To exclude that the loss of viability may be due to the shearing of filaments when plating, we measured viability using two different procedures in which mechanical spreading is not required. Either overlay in soft agar or measurement of the most probable number (MPN) using liquid cultures (16) detected a similar loss in the viability of cells when deprived from FtsZ (results not shown).

The loss of plating efficiency is not alleviated by the addition of exogenous catalase or the use of spent medium for the dilutions (not shown), suggesting that it is not caused by an increased sensitivity to peroxide radicals or by the fluctuation of quorum-sensing diffusible intermediates at low cell densities.

We also measured plating efficiency of PAT84, a strain harboring the thermosensitive *ftsZ84*(G105S) allele under control of its natural promoters (17). This mutation severely reduces GTP binding and hydrolysis and precludes FtsZ84 from forming protofilaments *in vitro* (18). Although a shift to the restrictive temperature reduces the viability of the thermosensitive PAT84 cells to levels not lower than 20%, viability of FtsZ-deprived VIP205 cells in the absence of IPTG at 42°C drops to nearly 1% (Fig. 1E), consistent with the values found at 37°C (Fig. 1D). These results suggest that in contrast to FtsZ depletion, the presence of the thermally inactivated FtsZ84 has a much lower impact on plating efficiency.

High salt concentration, but not sucrose, has a protective effect on PAT84, allowing the strain to grow at the restrictive temperature (18). To test if a similar protection could be exerted on FtsZ-deprived VIP205 cells, we plated them on LB medium without salt (0% NaCl) or LB containing high concentrations of salt (1% NaCl) or sucrose (6.5%). None of the additives had a protective effect on the VIP205 cells growing in the absence of the inducer (Fig. 1F). These results suggest that the low levels of FtsZ cannot be compensated for by these chemical protectants.

### FtsZ deprivation does not compromise the recovery of potential septation sites during growth in liquid medium.

To find if the loss of plating efficiency after FtsZ deprivation could be caused by irreversible damage to the potential septation sites within the VIP205 filaments, we allowed cells to recover the FtsZ levels required to sustain the normal rate of particle increase. We then examined if upon returning to balanced growth conditions, the filaments divided to produce the number of cells that had been missed due to the division block during deprivation.

The results show that upon readdition of IPTG (30 µM) to cultures maintained for 2 h in its absence, both the FtsZ levels and the number of FtsZ rings progressively recovered (Fig. 1A and B, respectively). FtsZ levels similar to those found in the unperturbed culture before FtsZ deprivation were attained at approximately 40 min after the readdition of IPTG (Fig. 1A). At this time, the morphology of the cells, although slightly longer than those in the unperturbed culture, was no longer filamentous (Fig. 1B; see Fig. S1A in the supplemental material). Sixty minutes after the readdition of IPTG, cell length was restored to the value shown by both the unperturbed culture and the parental strain, suggesting that division block could be reversed when the VIP205 cells were allowed to regain the FtsZ levels that they had prior to deprivation.

**FIG 2:**
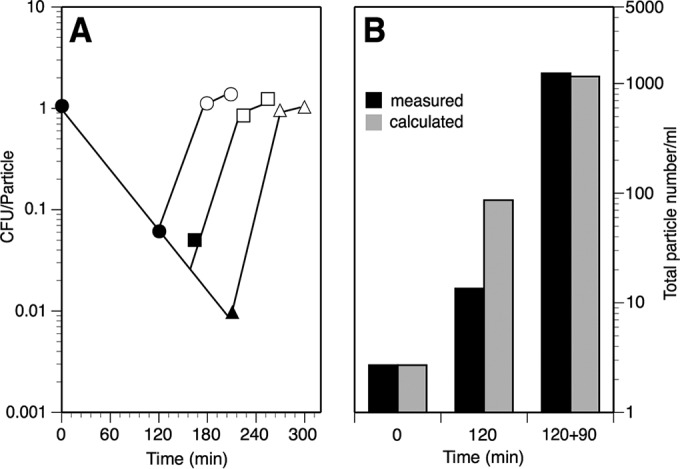
Recovery of viability and total cell number of VIP205 during restoration. (A) Plating efficiency of VIP205 following restoration of *ftsZ* transcription. A culture of VIP205 cells growing exponentially in the presence of 30 µM IPTG was transferred at time zero to medium without inducer (filled symbols). IPTG was added back to fractions from the FtsZ-deprived culture at 120 min (circles), 165 min (squares), and 210 min (triangles) to allow for the resumption of FtsZ synthesis, and these fractions were allowed to grow for three and six doubling times (open symbols) in the presence of the inducer. In each case, the number of CFU per particle was measured at the times indicated. (B) Total and calculated number of particles after deprivation or restoration of *ftsZ* transcription. Black bars show the number of particles measured in samples withdrawn at the times indicated. Gray bars correspond to the result calculated by multiplying the initial value at time zero by (2 × the number of doublings spent by each sample since the beginning of the experiment) + 1. The sample at 120 min had undergone 120 min of FtsZ deprivation in the absence of inducer, while the sample at 120 + 90 min had spent an additional 90 min of restoration after IPTG readdition. The measured value at time zero was 2.7 × 10^7^ particles ml^−1^.

Independently of the time spent under FtsZ deprivation (up to 210 min), readdition of IPTG to the deprived cultures allowed restoration of the plating efficiency upon growth in its presence for three doubling times (Fig. 2A). Moreover, the plating efficiency of these cultures was maintained even if 1/4 dilutions were made to keep their cell density at values able to sustain balanced growth, allowing us to further study their recovery under physiological conditions.

Restoration of the plating efficiency and attainment of the normal length when the FtsZ levels return to the values of the unperturbed culture (140% of the wild-type ones) may result from two alternative possibilities. In the first one, all of the potential septation sites within a filament become reactivated, so that all the filaments undergo successive divisions to originate a number of viable normal-length cells equal to the number of potential septation sites in the filament + 1 (i.e., cells with one septum produce two daughters, cells with three produce four daughters, etc.). The number of resultant cells should therefore be equal to the number of cells present in an unperturbed culture if they had been allowed to grow unrestrictedly during the same time. Conversely, if only a few septation sites within a filament regained their activity, they would produce a lower number of cells. Nevertheless, a population with a majority of cells with normal morphology could be generated assuming that only the short cells containing one active site could grow normally, diluting out the rest in which potential septation sites were not active. These would then remain as a residual proportion of nondividing filaments. In this case, the number of cells in the final population would be lower than the number present in an unperturbed culture if kept growing for an equal period.

To detect which scenario is correct, we measured the number of particles at three key time points during an experiment involving FtsZ deprivation and restoration. The values obtained at the end of the FtsZ restoration period were identical to the values that could be calculated from the initial particle number and the doubling time of VIP205 growing in the presence of the inducer (Fig. 2B). This agrees with the first alternative and demonstrates that the vast majority if not all of the potential sites within an FtsZ-deprived filament were reactivated during the restoration of FtsZ levels. In agreement with the reactivation of the old potential division sites, D’Ari and collaborators found that the inhibition of FtsZ by SulA caused a division block that could be quickly released by the proteolytic action of Lon on SulA (19). In this case, division occurred at the old potential septation sites. Selection of these sites for the reassembly of the new divisomes may be a consequence of the function of the septation site selection mechanisms—i.e., the Min and the Noc systems. In addition, the progression of the proto-ring into the mature divisome may involve a modification of peptidoglycan occurring as a penicillin-insensitive synthesis (12). Reactivation of septation at exactly the old sites after FtsZ restoration or reactivation may be favored if the peptidoglycan structure is permanently modified by this mechanism.

### FtsZ-deprived cells contain an abnormally high number of unsegregated nucleoids.

The absence of FtsZ prevents the assembly of other components of the divisome into the division ring, and among them is FtsK (4, 20), a protein that establishes a link between septation and nucleoid segregation participating in chromosome dimer resolution (reviewed in reference 18). Defects in nucleoid segregation have been reported when FtsK disassembles as a consequence of FtsN depletion (4). However, data previously reported by our laboratory failed to find a difference in segregation of the nucleoids within VIP205 filaments deprived of FtsZ (14).

**FIG 3:**
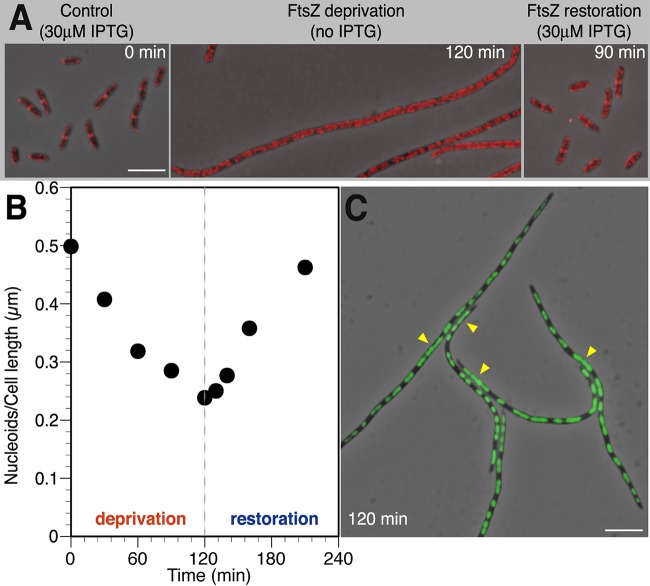
Effect of FtsZ deprivation and restoration on the localization of FtsK and on nucleoids. Cultures of VIP205 were grown as described in the legend to Fig. 1. At the indicated time points during FtsZ deprivation or restoration, cells were withdrawn, fixed with a mixture of methanol and acetic acid (4:1), and processed as shown in each panel. (A) Immunolocalization of the FtsK protein in VIP205 cells. FtsK protein (pseudocolored red) was visualized by immunofluorescence microscopy after staining with the MVC6 purified antibody. Overlaps of immunofluorescence images with phase-contrast images at the indicated times after FtsZ deprivation and subsequent FtsZ restoration are shown. The size bar represents 5 µm. (B) Nucleoid segregation in VIP205 cells undergoing FtsZ deprivation. The values correspond to the ratio of the total number of nucleoids over the total cell length of at least 100 cells at each sampled time. Nucleoids were revealed by SYTO9 staining and were considered to be distinct only if separated from the adjacent ones by a region with no fluorescence. (C) Representative fluorescence micrograph of VIP205 filaments at 120 min of FtsZ deprivation showing unsegregated nucleoids (arrows) in cells fixed as in panel A. The SYTO9-stained chromosomal DNA appears green on overlapping phase-contrast images. The size bar represents 5 µm.

To verify that FtsK is disassembled from the divisome when FtsZ is depleted in VIP205 in the absence of IPTG, we studied the localization pattern of FtsK in VIP205 cells at different time intervals during FtsZ deprivation. As expected, together with the depletion of FtsZ, we found that FtsK did not localize properly into rings in VIP205 cells when grown for 120 min without IPTG (Fig. 3A).

To investigate the impact of FtsZ depletion on nucleoid segregation, we utilized the nucleic acid stain SYTO9 and calculated the ratio of nucleoid number to cell length during FtsZ deprivation and restoration. Under control conditions, the nucleoid/cell-length ratio remained at 0.5 nucleoid/μm (Fig. 3B), suggesting that on average two daughter chromosomes are present per normal cell length (ca. 4 µm). In addition, while only 3% of the VIP205 cells grown with 30 µM IPTG exhibited defective nucleoid morphology, this number increased to 30% in the filaments produced after 120 min of FtsZ deprivation. In these FtsZ-deprived filaments, the missegregated chromosomes form a continuous mass (Fig. 3C, arrowheads), in contrast to the well-segregated ones that resemble prolate spheroids. In consequence, the nucleoid/cell-length ratio diminished to 0.23, less than half of the initial value (Fig. 3B).

To resolve the discrepancy between these results and those from our previous publication (14), we have compared the procedures used to obtain the data in the two cases. We found an effect of the fixation procedure (a mixture of methanol and acetic acid versus formaldehyde) on the shape of the nucleoid image. The comparison indicates that our former technology (formaldehyde) failed to evidence connections between unsegregated nucleoids, leading to a significant underestimation of the frequency of abnormal segregation (14).

Consistent with the result showing that potential septation sites are not damaged during FtsZ deprivation, we found that FtsK readily concentrates at the midcell, where septation sites are formed (Fig. 3A), and nucleoid number per cell length returns to the values of the unperturbed culture once FtsZ synthesis is restored (Fig. 3B).

### Changes in gene expression during FtsZ deprivation.

We find that failure to initiate the assembly of a divisome in the presence of low FtsZ levels affects nucleoid segregation and viability of *E. coli* VIP205 cells and modifies the assembly of divisome proteins such as FtsK. Expression of *ftsZ* is controlled by a large number of signals (21, 22), which led us to investigate if the effects of its absence, besides the obvious cessation of septation, may bring other global changes to the physiology of the cell.

Using quantitative real-time reverse transcription-PCR (qRT-PCR), we investigated if FtsZ depletion affects the expression of relevant genes involved in cell division, DNA transactions, protein synthesis, or global regulation. The quality and integrity of the RNA obtained from cultures grown in the absence of IPTG were tested by examining rRNA after electrophoretic separation (not shown). While the rRNAs from cultures depleted of FtsZ for a maximum of 120 min conserved integrity, the rRNA obtained from cultures in which FtsZ was depleted for longer periods showed signs of degradation precluding the analysis of transcript levels after longer deprivation periods. cDNA samples from three experimental replicas of VIP205 cultures grown with 30 µM IPTG (control samples), no IPTG for 120 min (FtsZ depletion), or 90 min after IPTG readdition (FtsZ restoration) were run simultaneously in the same assay, along with samples for a standard curve of each one of the genes analyzed.

The qRT-PCR data (see Table S1 in the supplemental material) confirmed that after 120 min of FtsZ deprivation, the transcription pattern of VIP205 cells shows several changes, coincident with their loss of resiliency. The most significant ones include the overexpression of genes responding to DNA damage (fold change: *alkB*, 16-fold; *mutH*, 4-fold; *mutS*, 9-fold; *obgE*, 13-fold; *phrB*, 15-fold; *recB*, 6-fold; *recC*, 7-fold; *seqA*, 8-fold; and *tus*, 4-fold), cell division (*ftsA*, 3-fold; *ftsQ*, 4-fold; *ftsK*, 2-fold; *zipA*, 2-fold; and *sulA*, 4-fold), or transcription factors (*dpiA*, 3-fold; *dipB*, 4-fold; and *stpA*, 25-fold). Transcription of several genes was repressed, including ribosomal genes (*rplQ*, <0.01-fold; *rpmF*, 0.01-fold) and global transcriptional regulators (*dksA*, 0.01-fold). The altered expression patterns of most of the genes analyzed can be correlated with the observed physiological changes that these cells undergo during FtsZ deprivation. The most significant decrease was found in *dksA*, whose product is a master regulator of transcription at multiple levels (reviewed in reference 23).

### Cells deprived of FtsZ do not accumulate DNA double-strand breaks.

We found that some genes that respond to DNA damage and more specifically those genes that are directly involved in the repair of double-strand breaks (DSBs)—e.g., *recB*, *recC* and *recD*—had an altered transcription pattern after FtsZ deprivation (see Table S1 in the supplemental material). We then tested if the loss of plating efficiency could derive from the generation of unrepaired DSBs after successive rounds of replication of the unsegregated nucleoids in VIP205. Null mutations in the *recA*, *recB*, or *lexA* genes have been described to prevent repair of DSBs leading to cell death (24, 25). VIP205 Δ*recA*, VIP205 *recB268*::Tn*10*, and VIP205 *lexA ind3* strains were constructed. None of these mutations affected the VIP205 growth rate during FtsZ deprivation and restoration, indicating that the absence of FtsZ does not induce lethal DSBs (see Fig. S2 in the supplemental material).

### Membrane integrity at low FtsZ levels becomes compromised.

In addition to the defects in chromosome segregation, the simple microscopic inspection of VIP205 filaments did not reveal other salient features that could help to explain their loss of viability. However, the measurement of transcript abundance under FtsZ deprivation suggests that the amounts of transcripts encoding cytoplasmic membrane proteins such as ZipA, FtsK, and DpiB increase (see Table S1 in the supplemental material). Moreover, direct quantification of amounts of protein indicated that the ZipA levels in the cytoplasmic membrane were increased (see Fig. S3 in the supplemental material). We have previously shown that high ZipA levels are deleterious and above certain values can disrupt the normal appearance of the cytoplasmic membrane and even cause shrinkage *in vitro* in a vesicle assay (26). We therefore decided to check if the loss of viability was accompanied by alterations in the membrane. We tested the functional integrity of the cytoplasmic membrane by staining FtsZ-deprived filaments with propidium iodide and quantifying red-stained cells by flow cytometry. Healthy membranes are refractory to propidium iodide penetration, whereas damaged ones allow the entry of the dye.

**FIG 4:**
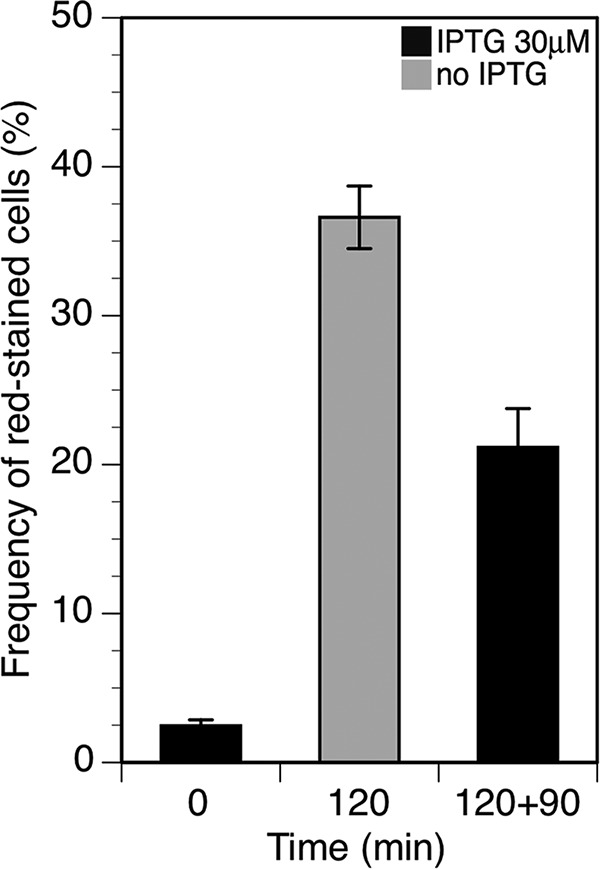
Effect of FtsZ deprivation and restoration on the integrity of the membrane. Shown is the frequency of cells that become stained by propidium iodide after deprivation or restoration of *ftsZ* transcription when grown as described in the legend to Fig. 1. Samples were withdrawn at the times indicated, and the fluorescence of 10^5^ cells was measured using flow cytometry. Error bars represent the standard deviations from at least three independent experiments.

Flow cytometry distributions (Fig. 4) show an increase in the number of cells that stained red in FtsZ-depleted populations indicating that membrane integrity is compromised. After 90 min following FtsZ restoration, the frequency of cells that stain red diminishes, and plating efficiency is recovered, as described above (Fig. 2A). However, a residual number of cells (20%) do not become refractory to propidium iodide stain, suggesting that the membrane takes a longer time than plating efficiency to return to the unperturbed conditions.

### Reversion of changes during the recovery of the normal FtsZ levels.

The global transcription pattern of VIP205 cells obtained from cultures that were maintained for 120 min in the absence of IPTG and then allowed to recover during 90 min after the addition of the inducer (30 µM) was also compared with the pattern of the unperturbed culture. After recovery, the levels of *ftsZ* transcript and product, the abundance of FtsZ rings, and the particle doubling time all reached values similar to those found in VIP205 growing in the presence of 30 µM IPTG (Fig. 1; see Table S1 in the supplemental material). Chromosome segregation and membrane integrity (Fig. 3 and 4, respectively) were also recovered 90 min after IPTG was added back to the culture.

However, the global expression pattern during the recovery of the FtsZ normal concentration showed several differences in the transcript levels of specific gene groups compared to the unperturbed culture. In particular, the abundance of the ribosomal *rpmF* and *rplQ* and the global transcription regulator *dksA* transcripts failed to regain transcript levels identical to those found in the unperturbed culture. Some genes whose transcript levels were induced in the absence of FtsZ remained at high levels after FtsZ restoration. These genes include *clpP*, coding for a protease that degrades FtsZ (15), a set of genes related to DNA damage (*mutH mutS*, *phrB*, *recB*, *recC*, *umuC*, and *umuD*), and three genes involved in the initiation or termination of chromosome replication (*obgE*, *seqA*, and *tus*).

## DISCUSSION

Decreasing the pool of FtsZ molecules to values below 20% of the initial level in VIP205 cells (Fig. 1A and C) blocks division and promotes filamentation (Fig. 1B; see Fig. S2 in the supplemental material). FtsZ deprivation is associated with additional unexpected defects, including an increased number of improperly segregated nucleoids (Fig. 3), changes in global transcription, some of them pleiotropic (see Table S1 in the supplemental material), and a failure in membrane function (Fig. 4). We conclude that the combination of these changes weakens the cell, diminishing its resiliency when stressed further during the plating procedure, causing a loss of plating efficiency on solid medium containing 30 µM IPTG, in contrast with the return to growth observed upon the addition of 30 µM IPTG to liquid cultures (Fig. 1D). Restoration of the initial FtsZ levels in liquid cultures allows the recovery of the division activity at almost all of the potential septation sites present in the filaments (Fig. 2B) and largely reverts the other effects.

### Chromosome segregation and the absence of FtsZ.

**FIG 5:**
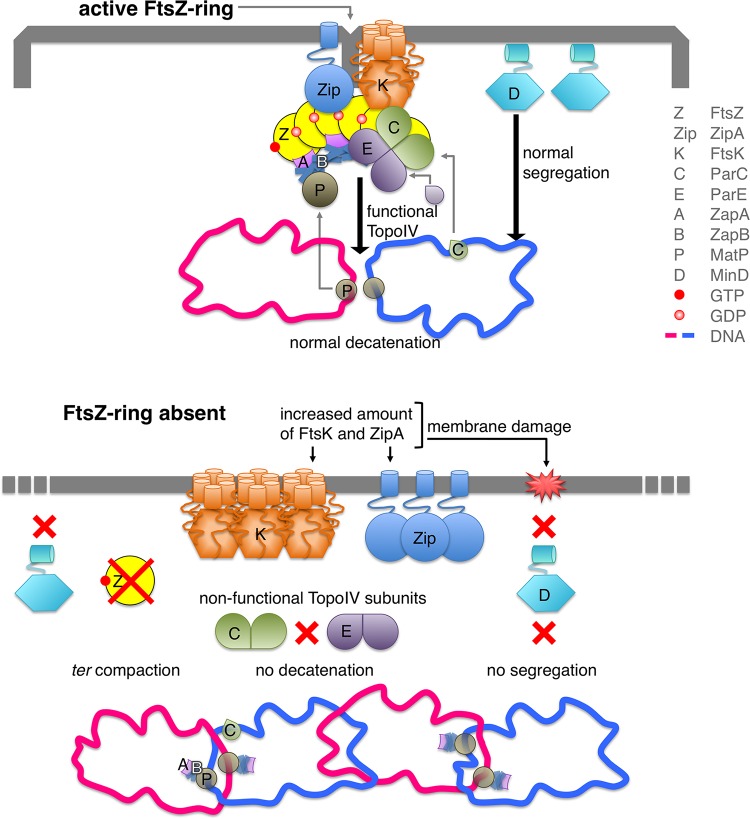
Diagram of the links showing the connections between the deprivation of FtsZ and the decrease of the resiliency of VIP205 cells. In the top panel, when FtsZ is produced at normal concentrations, ZipA, FtsK, and MinD are maintained at levels that support division, nucleoid segregation, and recombination. In the bottom panel, when the pool of FtsZ molecules is depleted the free ZapB might interact with MatP reinforcing the compaction of the *ter* region and delaying the normal segregation of the daughter chromosomes. Mislocalization of FtsK might favor the formation of catenanes, as a functional Topo IV would not assemble at the septum, affecting chromosome segregation. Moreover, the number of ZipA and FtsK molecules inserted into the membrane of the filaments is increased, affecting the functional integrity of the membrane. This prevents the normal attachment of MinD and thus its function in chromosomal segregation, which may add to the inefficient segregation of the nucleoid.

Our results in Fig. 3 suggest that the assembly and correct localization of a functional Z-ring have a role in nucleoid segregation. FtsK is one of the divisome components that do not localize properly in the absence of FtsZ. It is a bitopic protein that binds to the membrane and to specific DNA sequences linking nucleoid segregation and septation (20, 27). In normal division events, FtsK localizes before constriction as part of the divisome at midcell (Fig. 5, top row), where MatP facilitates FtsK-catalyzed ordered segregation of Ter loci by maintaining the Ter domain cohesion (28). MatP interacts at midcell with the FtsZ accessory protein ZapB to stabilize the FtsZ bundles (29) and with the *ter* region of the newly replicated chromosomes (28). At a later stage, FtsK removes MatP from the *ter* region and promotes the ordered segregation of the daughter chromosomes in the majority of cells (28). In the absence of the FtsZ ring (Fig. 5, bottom row), FtsK is spread at the membrane along the cell, and therefore it is not at the correct position to displace MatP and to process the sister chromosomes to allow normal segregation. Moreover, the absence of FtsZ may direct the ZapB protein toward MatP and therefore reinforce the compaction of the *ter* region, delaying normal chromosome segregation (28). In the absence of FtsK localization, a functional topoisomerase IV (Topo IV) cannot be assembled at midcell (30), precluding proper nucleoid splitting (Fig. 5, bottom row) (31).

MinD, the ATPase component of the FtsZ ring-positioning MinCDE complex (reviewed in reference 32), also contributes to establish a connection between chromosome dynamics and the membrane. Altering the levels of MinCDE proteins either by up- or downregulating the transcription of the *minB* operon impairs nucleoid segregation in *E. coli* (33). Moreover, the membrane-associated MinD binds nonspecifically to DNA and transiently tethers the newly replicated chromosomes to the membrane (34). Association of MinD with the membrane through its MTS domain is damaged when the membrane potential is lost (35), as happens during FtsZ deprivation. At the same time, the levels of *minD* transcripts are also reduced under these conditions (see Table S1 in the supplemental material). Both effects may thus exacerbate the defects in chromosome segregation observed in VIP205 filaments (Fig. 5, bottom row). Our results (see Fig. S2 in the supplemental material) show that the unsegregated nucleoids contain no major DNA damage as segregation recovery does not require the presence of RecA, RecB, or LexA once the FtsZ levels are restored (see Fig. S2).

### Transcriptional changes in the absence of FtsZ.

FtsZ-deprived cells show major changes affecting the transcripts of genes involved in chromosome segregation, cell envelope, transcription, and translation (see Table S1). The analysis of these changes outlines a global view of the state of cells undergoing FtsZ deprivation and, in the absence of a defined cause, allows us to speculate on a sequence of events leading from the loss of FtsZ to the loss of plating efficiency.

A synergistic effect on nucleoid segregation can result from the overexpression of *seqA*, *obgE*, *minE*, *dpiA*, and *dpiB*. High levels of SeqA inhibit the action of topoisomerase IV (36), whereas large amounts of ObgE impair partitioning of daughter chromosomes after a replication round (37). Overexpression of *minE* also impairs the correct segregation of nucleoids (38). DpiA and -B proteins, which make up a two-component signal transduction system that regulates transcription (39), interfere with DNA replication and segregation of both the *E. coli* chromosome and pSC101 derivatives (39, 40).

Regulation of the transcription of genes whose products participate in the normal structure of the bacterial envelope can be affected by changes in the architecture of the chromosome mediated by *hns* and *stpA*, two transcriptional dual regulators also overexpressed in FtsZ-depleted cells (41). Among them are the expression patterns of *ompC* and *ompN*, encoding outer membrane proteins (OMPs), and of several genes, such as the *rfa* operon, encoding enzymes that participate in lipopolysaccharide synthesis, or *gmd* and *wzc*, encoding enzymes for capsular polysaccharides synthesis (see reference 42 and references therein).

FtsZ-deprived cells also suffer defects in the translation and transcription machineries, likely contributing to rendering the cells unable to quickly respond to stress. A decrease in the transcripts of most of the components of the ribosomes, particularly severe in the case of *rpmF* and *rplQ*, is likely to affect ribosomal rejuvenation during FtsZ deprivation. These damages may be aggravated by defects in transcript synthesis imposed by the low levels of *dksA* messenger (23). DksA positively or negatively modulates the RNA polymerase elongation activity, and its absence leads to uncoupling the production of ribosomes from the cellular demand for protein synthesis (43).

The restoration of *ftsZ* transcription is accompanied by the recovery of most of these changes affecting the transcription and translation machinery. Even if this recovery is not total for some specific genes, such as *seqA*, *obgE*, *rpmF*, *rplQ*, and *dksA*, it is sufficient to allow the full recovery of the normal rate of mass increase and the reactivation of the potential septation sites accumulated during deprivation. The restoration of FtsZ levels finally results in cell proliferation and plating efficiency values returning to normal.

### Envelope integrity in filaments deprived of FtsZ.

Our data (see Table S1 in the supplemental material) show that upon FtsZ depletion the amounts of inner membrane proteins, including several divisome components, ZipA (see Fig. S3 in the supplemental material) (25), FtsA, FtsQ (44, 45), and FtsK, increase.

Overexpression of the transcriptional dual regulators *hns* and *stpA* (see Table S1) may in addition lead to altered levels of some outer membrane proteins (OMPs), such as OmpC or OmpN, and proteins that participate in the synthesis of flagella, such as FlhA or FliC, which contribute to modification of the cell envelope (described above and see reference 42). The disruption of the cytoplasmic membrane proton motive force following FtsZ deprivation can also affect the translocation of several OMPs (46) and thus modify the outer membrane composition of VIP205 filaments. These alterations in the cell envelope lead to an increased number of filaments that allow permeation of propidium iodide, contributing to the loss of *E. coli* viability in the absence of FtsZ.

We find that upon recovery of normal FtsZ levels, a residual number (20%) of propidium iodide-positive cells remain. This apparent contradiction may result from the mosaic structure of the membrane. Upon restoration, some defective and therefore permeable patches may remain until the total membrane surface is completely replaced or diluted by growth.

### The steady production of FtsZ maintains viability.

We find that the absence of FtsZ has severe effects on nucleoid segregation, on many relevant transcripts, and on the integrity of the membrane, culminating in a reduced resilience that diminishes plating efficiency when deprived filaments are confronted with changes in their environment. In contrast, blocking cell division by overproduction of the FtsZ inhibitor SulA, without altering the levels of FtsZ, causes no major changes in the *E. coli* transcriptome when measured using a GeneChip *E. coli* antisense genome array (47). These arrays failed in our hands to detect significant changes of transcript levels, even for the *ftsZ* gene, in VIP205 in the absence of IPTG (not shown). Moreover, inhibition of FtsZ activity by SulA does not modify the FtsZ pool, and the cells can resume septation immediately after cessation of SulA action because the FtsZ protein may reassemble at potential division sites upon cleavage of SulA by the Lon protease (18). Different from those studies, our results from VIP205 were obtained when the amount of FtsZ was substantially lower. We postulate that the presence of an FtsZ protein, even when not active, makes the cells more resistant to environmental changes.

Our results obtained with PAT84 agree with this hypothesis. At the restrictive temperature, these cells steadily produce almost normal amounts of FtsZ84 (Fig. 1E), but the GTPase activity and bundling of FtsZ84 are reduced (18). PAT84 cells can divide normally at the permissive temperature but not at the restrictive temperature, unless suppressed by high salt concentrations (Fig. 1F). The plating efficiency of PAT84 was not greatly affected even when grown for several generations under conditions in which the amount of FtsZ84 remained at normal levels but its activity was lost. This contrasts sharply with the decrease in viability observed in VIP205 cells when their FtsZ levels are low enough to fail to assemble into FtsZ rings (Fig. 1E).

Gene expression of the “division and cell wall” cluster, in which *ftsZ* is located, is regulated by a complex set of signals, including a wealth of promoters to transcribe *ftsZ* (21, 22, 48). This abundance of regulators is perhaps not surprising when considering that the absence of the FtsZ protein alone, besides blocking division, has such profound effects on the physiology and plating efficiency of *E. coli* cells.

Our results highlight an essential novel role of the FtsZ protein besides its already known effect in the constriction of *E. coli*, namely, its requirement to maintain viability of the nondividing cells. Filamentation in the absence of division, as in the case of FtsZ inhibition by SulA after DNA damage, may be part of a survival strategy in which the induction of the SOS response allows cells to recover viability (19). However, in the absence of FtsZ, the *E. coli* filaments enter into a fragile equilibrium in which additional injuries, as a high dilution, are not tolerated. This is probably a pleiotropic effect in which following FtsZ deprivation, the cell envelope becomes affected, losing its integrity (Fig. 4). This behavior is reminiscent of the lysis induction observed in *S. pneumoniae* following a cessation of growth (6) and may be exploited to design effective antimicrobial compounds. Construction in the test tube, as proposed by bottom-up synthetic biology, of a simplified cell division mechanism in which some of the components of the divisome are absent may need to supply or substitute for some of the presently unsuspected roles of the missing parts to maintain the integrity or viability of the resulting construct.

## MATERIALS AND METHODS

### Strains and growth conditions.

For details of the strains and growth conditions used in this study, see Text S1 in the supplemental material.

### Cell parameter determination.

Optical density at 600 nm was measured using a Biowave CO8000 spectrophotometer. For particle number measurements, aliquots were fixed in 0.75% formaldehyde and counted using a ZM Coulter counter with a 30-µm orifice connected to a C1000 Channalyzer from Coulter Electronics.

Damage to membrane integrity was checked by measuring with flow cytometry the frequency of cells that were able to stain red with propidium iodide (26).

To determine the frequency of nucleoids per unit of cell length, the same fields were first photographed in the phase-contrast mode of an Olympus BX61 microscope fitted with a 100× immersion oil lens, where the cell length is quantified with the ImageJ plug-in ObjectJ and then with the fluorescein isothiocyanate (FITC) filter (U-MNIB2; excitation, 470/20; emission, 515 nm; beamsplitter 505) to localize and count the SYTO9-stained chromosomes.

### Viability measurements.

Cells were maintained at exponential balanced growth under permissive conditions and transferred to nonpermissive conditions before plating of suitable dilutions. VIP205 cells were spotted onto LB containing 50 µg ml^−1^ kanamycin and 30 µM IPTG at 37°C. PAT84 cells were spotted onto Nutrient Broth supplemented with 50 µg/ml thymine (NBT) at 30°C.

To measure VIP205 cell viability in liquid medium, 10-fold dilution series of cell cultures were done under the three different conditions, in order to get one viable count per milliliter. Assuming a Poisson distribution of the viable cells in the inoculated tubes, using the MPN tables (16), the number of viable cells in the culture was calculated.

### Immunofluorescence microscopy.

Exponentially growing cells were prepared to immunolocalize division proteins (4). Samples were incubated overnight at 4°C with a 1:200 dilution of the anti-FtsZ rabbit polyclonal antibody MVJ9 or with a 1:50 dilution of the anti-FtsK rabbit polyclonal antibody MVC6 (4). To detect FtsZ, a Cy3-conjugated secondary antibody (Amersham Pharmacy Biotech) was used at a 1:100 dilution, and to detect FtsK, an Alexa-594-conjugated secondary antibody (Molecular Probes) was used at a 1:500 dilution. The software used for image grabbing was AnalySIS (Soft Imaging System GmbH) with Adobe Photoshop 7.0 or CS for image processing.

### Immunoblotting.

Exponential cultures of *E. coli* VIP205 were collected at an optical density of 600 nm of 0.2 to 0.3. Samples corresponding to 0.1 OD_600_ unit were separated by SDS-PAGE and revealed after Western blotting with MVJ9 (anti-FtsZ) and MVJ4 (anti-EF-Tu) purified polyclonal antibodies (49) and protein A coupled to peroxidase (Bio-Rad).

## SUPPLEMENTAL MATERIAL

Text S1  Supplemental materials and methods. Download Text S1, DOC file, 0.1 MB

Figure S1  Cell length and mass increase after deprivation and restoration of *ftsZ* transcription. Cultures of VIP205 cells were grown as described in the legend to Fig. 1 and sampled at indicated times during FtsZ deprivation and restoration. In the left panel (A), the length of 100 cells at each time point was quantified with the ImageJ plug-in ObjectJ. In the right panel (B), the mass increase of cultures of VIP205 or its parental strain was followed by measuring optical density units at 600 nm at the times indicated. Download Figure S1, TIF file, 7.6 MB

Figure S2  Mass increase of VIP205 derivative strains defective in DNA repair after deprivation and restoration of *ftsZ* transcription. Cultures of VIP205, VIP205 Δ*recA*, VIP205 *recB268*::Tn*10*, or VIP205 *lexA ind3* cells were grown as described in the legend to Fig. 1 and sampled at the indicated times during FtsZ deprivation and restoration. Mass increase of the cultures was followed by measuring optical density units at 600 nm. Download Figure S2, TIF file, 6.3 MB

Figure S3  ZipA protein levels after deprivation and restoration of *ftsZ* transcription. (A) Immunoblot showing cellular levels of ZipA in VIP205 cells after 120 min of *ftsZ* transcription deprivation and during restoration of *ftsZ* transcription. Samples were withdrawn at the times indicated. (B) Quantification of the amounts of ZipA during the experiment shown in panel A. The levels of the protein were made relative to the amount present at time zero. Download Figure S3, TIF file, 5.5 MB

Table S1  Relative expression levels as determined by qRT-PCR. Table S1, DOCX file, 0.1 MB
