# Supplementary material for: Life without Division: Physiology of Escherichia coli FtsZ-Deprived Filaments
Source: mBio. 2016 Oct 11;7(5):e01620-16. doi: 10.1128/mBio.01620-16 (PMC5061873; doi:10.1128/mBio.01620-16)
Supplement: Text S1 — Supplemental materials and methods. Download [file mbo005163022s1.docx]

**Supplemental Materials and Methods**

**Strains and growth conditions.**

Strain MC1061 (*F-, ∆(ara-leu)7697, [araD139]B/r, ∆(cod-lacI)3, galK16, galE15, l-, e14-, mcrA0, relA1, rpsL150(strR), spoT1, mcrB1, hsdR2(r-m+)*; Casadaban MJ, Cohen SN. J Mol Biol 138:179-207, 1980.), a wild-type strain for *ftsZ*, was routinely grown at 37ºC in Luria Broth (LB) Lennox.

Strain VIP205 (MC1061, *kan, Ptac::ftsZ*; Garrido T, *et al.,* EMBO J 12:3957-3965, 1993.) was grown at 37ºC in LB medium supplemented with 50 μg ml^-1^ kanamycin and 30μM IPTG. At this IPTG concentration cell size is similar to the parental strain.

VIP205 ∆*recA*, VIP205 *recB268* and VIP205 *lexAind3* repair-deficient strains were obtained by P1 transduction from JJC275 (AB1157 ∆(*recA*-*srl*)::*Tn10* [mini F-RecA+]); JJC777 (JJC40 *recB268*::*Tn10* [pDWS2]), or JJC443 is *lexAind3 malF::Tn10* (kind gifts from B. Michel), respectively. These strains were grown as VIP205.

Strain PAT84 (*F-, thr-1, leuB6(Am), ftsZ844(ts), fhuA2::IS2, lacY1, glnX44(AS), λ-, trp-1, hisG1, rfbC1, thyA719, malT1(λR), xyl-7, mtlA2, ΔargH1, rplL9(L?), thiE1, deo-73*; Lutkenhaus J, Wu H. J Bacteriol 143:1281-1288, 1980.) was grown in NBT (Nutrient broth No.2; Oxoid) supplemented with 50 mg ml^-1^ thymine, at 30ºC or non-permissive conditions of 42ºC.

**Viability measurements**

To measure VIP205 cell viability in liquid medium, 10-fold dilution series of cell cultures were done in triplicate, in order to get one viable count per ml. The number of viable cells in the culture was calculated assuming a Poisson distribution of the viable cells in the inoculated tubes using the MPN tables (Koch AL., p 179-207. *In* Gerhardt P, *et al.* (ed), Manual of Methods for General Bacteriology. 1981.).

**Quantitative real time RT-PCR.**

mRNA was isolated from three independent biological replicate cultures at indicated time points (0 min or 120 min for cultures without IPTG, and 90 min after IPTG readdition) with the RNAqueous RNA extraction kit (Ambion). cDNA was synthesised with the First strand cDNA synthesis kit (Amersham Biosciences). Quantitative real time qRT-PCRs reaction mixtures (25 μl) contained 12.5 μl of SYBR Green PCR master mix (Applied Biosystems), 10μM of each specific oligonucleotide primer, and 15.6 ng of cDNA. Quantitative PCR analyses were done on an ABI PRISM 7300 thermocycler (Applied Biosystems).

The primer pairs used for each gene were designed with the Primer Express 3.0 program (ABI Prism) and are available upon request. Fluorescence due to the binding of the SYBRgreen fluorochrome to double-stranded DNA was measured once per cycle. A standard curve was made for each amplicon using 10-fold dilution series of the cDNA in order to quantify the relative expression of each gene in the control and experimental conditions. cDNA from the experimental and reference samples were amplified in triplicate using amounts within the linear range of the standard curve. A melting curve confirmed the amplification of a single product. Results were normalized relative to those obtained for the *gapA* (D-glyceraldehyde 3-phosphate dehydrogenase) housekeeping gene, as its expression was not affected by inactivation of *ftsZ*.
