# Supplementary figures and images for: Life without Division: Physiology of Escherichia coli FtsZ-Deprived Filaments
Source: mBio. 2016 Oct 11;7(5):e01620-16. doi: 10.1128/mBio.01620-16 (PMC5061873; doi:10.1128/mBio.01620-16)

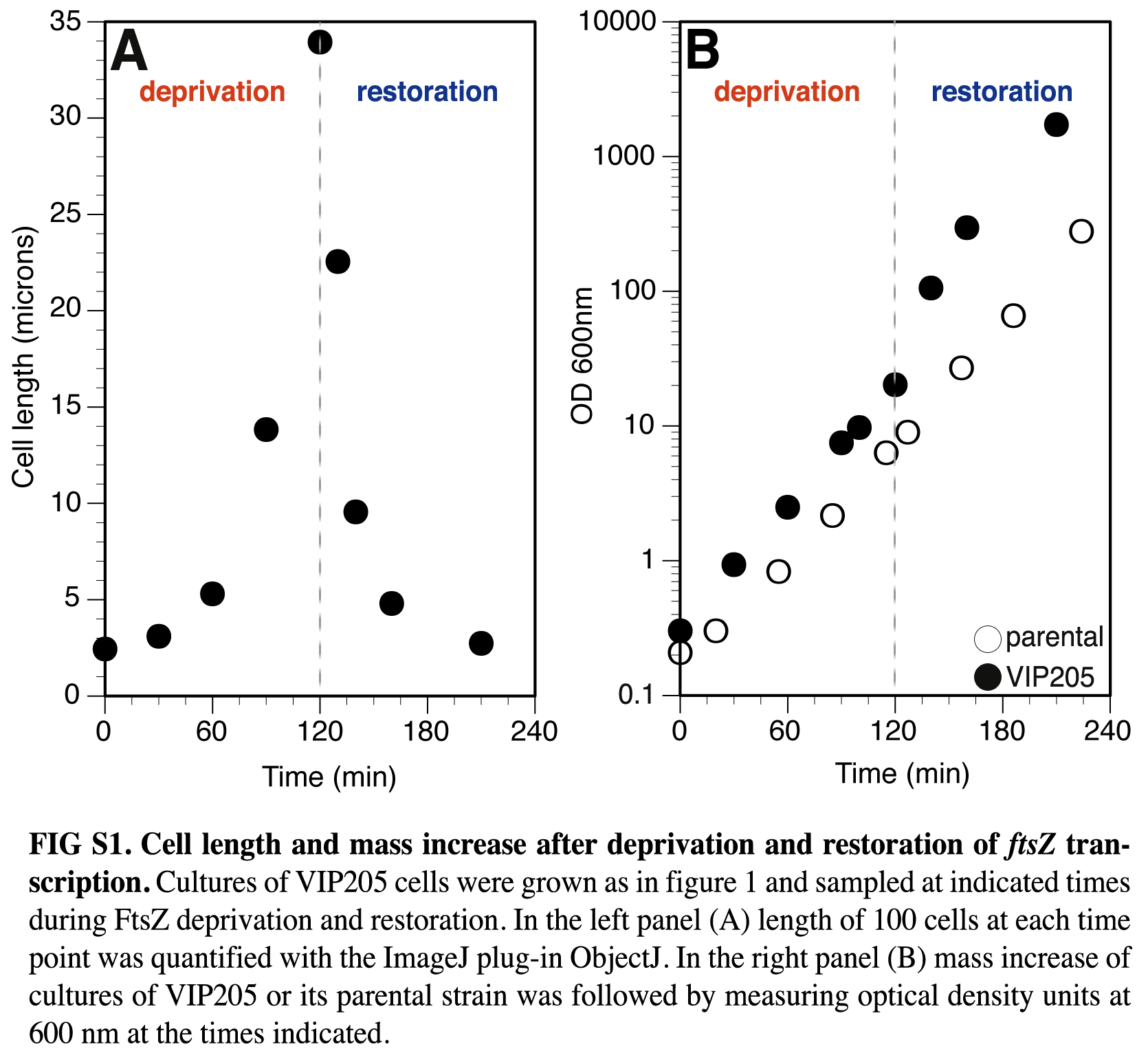

Supplement: Figure S1 — Cell length and mass increase after deprivation and restoration of ftsZ transcription. Cultures of VIP205 cells were grown as described in the legend to Fig. 1 and sampled at indicated times during FtsZ deprivation and restoration. In the left panel (A), the length of 100 cells at each time point was quantified with the ImageJ plug-in ObjectJ. In the right panel (B), the mass increase of cultures of VIP205 or its parental strain was followed by measuring optical density units at 600 nm at the times indicated. Download [file mbo005163022sf1.tif]

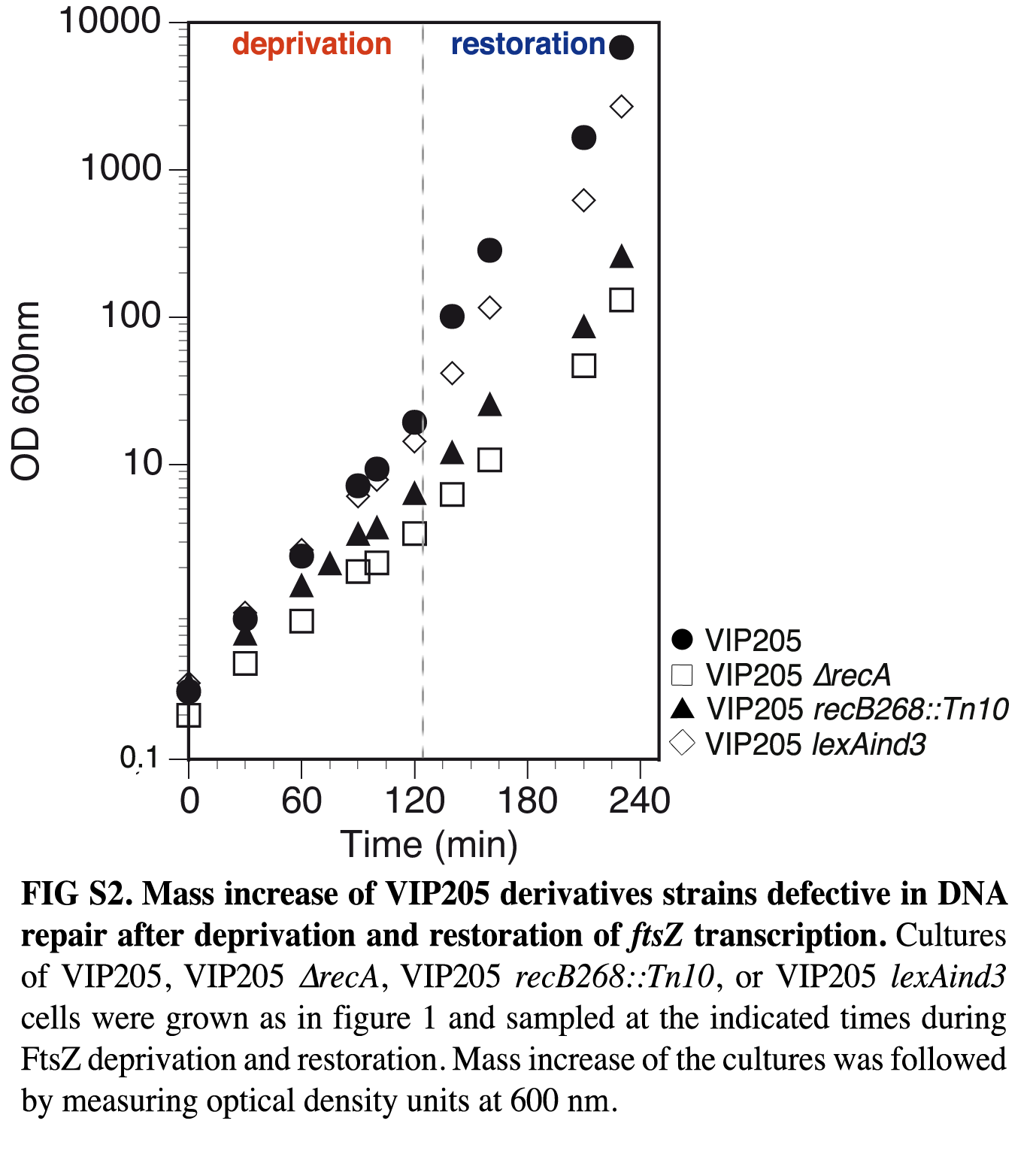

Supplement: Figure S2 — Mass increase of VIP205 derivative strains defective in DNA repair after deprivation and restoration of ftsZ transcription. Cultures of VIP205, VIP205 ΔrecA, VIP205 recB268::Tn10, or VIP205 lexA ind3 cells were grown as described in the legend to Fig. 1 and sampled at the indicated times during FtsZ deprivation and restoration. Mass increase of the cultures was followed by measuring optical density units at 600 nm. Download [file mbo005163022sf2.tif]

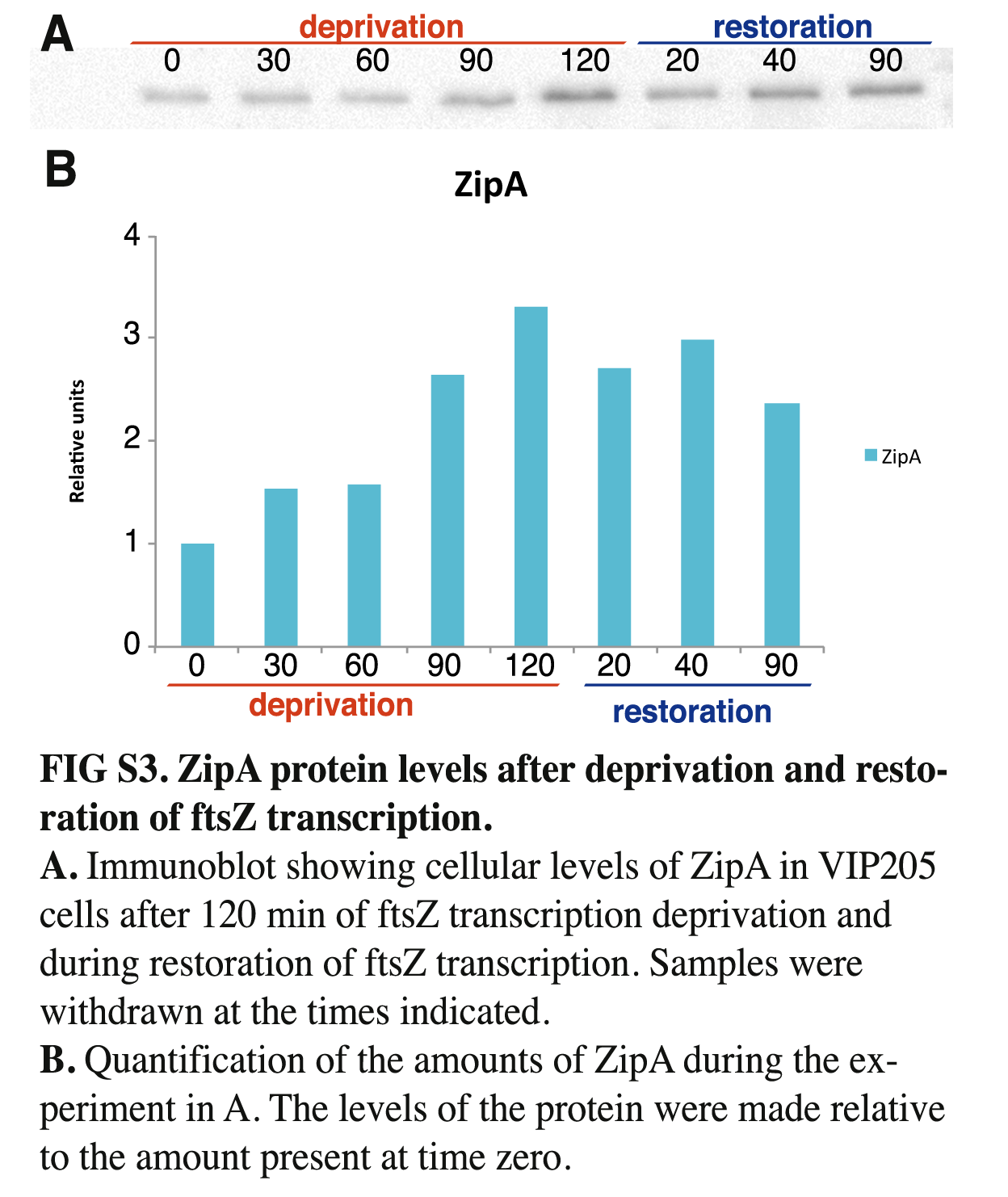

Supplement: Figure S3 — ZipA protein levels after deprivation and restoration of ftsZ transcription. (A) Immunoblot showing cellular levels of ZipA in VIP205 cells after 120 min of ftsZ transcription deprivation and during restoration of ftsZ transcription. Samples were withdrawn at the times indicated. (B) Quantification of the amounts of ZipA during the experiment shown in panel A. The levels of the protein were made relative to the amount present at time zero. Download [file mbo005163022sf3.tif]
