# Supplementary material for: Life without Division: Physiology of Escherichia coli FtsZ-Deprived Filaments
Source: mBio. 2016 Oct 11;7(5):e01620-16. doi: 10.1128/mBio.01620-16 (PMC5061873; doi:10.1128/mBio.01620-16)
Supplement: Table S1 — Relative expression levels as determined by qRT-PCR. [file mbo005163022st1.docx]

**Table S1.** Relative expression levels as determined by qRT-PCR

| Functional group and gene | | FtsZ | | | |  |
| --- | --- | --- | --- | --- | --- | --- |
|  |  | deprivation | | restoration | | EcoCyc product description |
|  |  |  |  |  |  |  |
| Cell division and morphogenesis | | | |  |  |  |
| b0083 | *ftsL** | 2.40 ± | 1.16 | 2.21 ± | 0.20 | Essential cell division protein |
| b0084 | *ftsI** | 2.65 ± | 1.17 | 0.75 ± | 0.20 | Essential cell division protein; penicillin-binding protein 3, transpeptidase involved in septal peptidoglycan synthesis |
| b0089 | *ftsW** | 2.52 ± | 1.36 | 1.79 ± | 0.54 | Essential cell division protein, membrane protein involved in stabilizing FstZ-ring during cell division |
| b0093 | *ftsQ** | 4.34 ± | 0.38 | 0.91 ± | 0.25 | Essential cell division protein, membrane anchored protein involved in growth of wall at cell septum |
| b0094 | *ftsA** | 3.15 ± | 0.70 | 1.33 ± | 0.36 | ATP-binding essential cell division protein involved in recruitment of FtsK to FtsZ-ring |
| b0095 | *ftsZ** | 0.42 ± | 0.21 | 1.69 ± | 0.03 | GTP-binding tubulin-like essential cell division protein |
| b0890 | *ftsK** | 2.96 ± | 0.88 | 1.20 ± | 0.41 | Essential cell division protein, couples cell division and chromosome segregation |
| b1174 | *minE* | 2.61 ± | 0.11 | 2.00 ± | 0.10 | Cell division topological specificity factor, inhibitory component of the MinCDE system that regulates septum placement, inhibitor of MinC |
| b1175 | *minD* | 0.75 ± | 0.20 | 0.92 ± | 0.26 | Membrane ATPase of the MinCDE system |
| b1176 | *minC* | 1.82 ± | 0.17 | 1.60 ± | 0.17 | Cell division inhibitor, component of the MinCDE and DicB-MinC systems |
| b2412 | *zipA* | 2.23 ± | 0.93 | 0.93 ± | 0.22 | Essential cell division protein, stabilizes FtsZ protofilaments |
| b2748 | *ftsB* | 2.87 ± | 0.86 | 1.42 ± | 0.15 | Essential cell division protein |
| b3017 | *sulA** | 4.53 ± | 1.82 | 4.61 ± | 1.53 | Inhibitor of FtsZ-ring formation and cell division |
| b3933 | *ftsN* | 1.49 ± | 0.16 | 1.45 ± | 0.38 | Essential cell division protein |
|  |  |  |  |  |  |  |
|  |  |  |  |  |  |  |
|  |  |  |  |  |  |  |
| DNA replication, modification, recombination and repair | | | | | | |
| b0687 | *seqA* | 8.76 ± | 1.88 | 7.78 ± | 0.84 | Negative modulator of initiation of replication |
| b0708 | *phrB** | 15.94 ± | 2.54 | 14.08 ± | 2.27 | Deoxyribodipyrimidine photolyase (photoreactivation) |
| b1183 | *umuD** | 1.78 ± | 0.64 | 3.18 ± | 0.30 | DNA polymerase V component involved in SOS mutagenesis and error-prone repair, processed to UmuD’ |
| b1184 | *umuC** | 1.06 ± | 0.17 | 2.22 ± | 0.20 | DNA polymerase V component involved in SOS mutagenesis and error-prone repair |
| b1610 | *tus* | 4.71 ± | 1.76 | 3.47 ± | 0.42 | DNA-binding protein; inhibition of replication at *Ter* sites |
| b1913 | *uvrC* | 0.98 ± | 0.51 | 1.18 ± | 0.26 | UvrABC Nucleotide Excision Repair Complex, repair of UV damage to DNA. |
| b1960 | *vsr* | 1.37 ± | 0.47 | 1.54 ± | 0.60 | DNA mismatch endonuclease of the very short patch (VSP) mismatch repair pathway |
| b2212 | *alkB* | 16.56 ± | 0.14 | 1.41 ± | 0.74 | Repair system for alkylated DNA and RNA |
| b2699 | *recA** | 2.04 ± | 0.33 | 1.23 ± | 0.02 | DNA strand exchange and recombination protein with protease and nuclease activity |
| b2733 | *mutS** | 9.45 ± | 1.83 | 12.71 ± | 1.92 | Component of the MutHLS complex, the methyl-directed mismatch repair pathway |
| b2819 | *recD** | 2.16 ± | 0.48 | 1.17 ± | 0.57 | DNA helicase, ATP-dependent dsDNA/ssDNA exonuclease V subunit, ssDNA endonuclease |
| b2820 | *recB** | 6.26 ± | 0.06 | 7.45 ± | 0.50 | DNA helicase, ATP-dependent dsDNA/ssDNA exonuclease V subunit, ssDNA endonuclease |
| b2822 | *recC** | 7.40 ± | 0.96 | 6.84 ± | 1.28 | DNA helicase, ATP-dependent dsDNA/ssDNA exonuclease V subunit, ssDNA endonuclease |
| b2831 | *mutH** | 4.30 ± | 0.32 | 6.84 ± | 1.39 | dGATC endonuclease in the MutHLS complex |
| b2894 | *xerD** | 1.40 ± | 0.31 | 1.39 ± | 0.71 | Site-specific recombinase, component of the XerCD system |
| b3183 | *obgE* | 13.24 ± | 1.98 | 19.65 ± | 1.09 | GTPase involved in DNA replication and ribosome assembly |
| b4170 | *mutL** | 1.44 ± | 0.45 | 1.37 ± | 0.56 | Component of the MutHLS complex |
|  |  |  |  |  |  |  |
| Transcription and translation regulation | | | | | |  |
| b0145 | *dksA* | 0.01 ± | 0.00 | 0.30 ± | 0.42 | RNA polymerase-binding transcription factor, translational regulator of *rpoS* |
| b0435 | *bolA* | 1.72 ± | 0.08 | 1.76 ± | 0.02 | Transcriptional dual regulator, cell morphology |
| b0619 | *dpiB* | 4.15 ± | 2.43 | 1.80 ± | 0.57 | Sensory histidine kinase in two-component regulatory system with DpiA |
| b0620 | *dpiA* | 3.91 ± | 1.88 | 1.65 ± | 0.51 | DNA binding transcriptional dual regulator |
| b1237 | *hns* | 2.80 ± | 0.15 | 1.26 ± | 0.44 | Transcriptional dual regulator |
| b1916 | *sdiA* | 1.82 ± | 0.55 | 0.37 ± | 0.03 | Transcriptional dual regulator of genes involved in cell division |
| b2669 | *stpA* | 25.68 ± | 4.14 | 0.78 ± | 1.10 | H-NS-like DNA-binding protein with RNA chaperone activity |
| b2741 | *rpoS* | 1.51 ± | 0.43 | 1.75 ± | 0.51 | RNA polymerase, sigma S-subunit |
|  |  |  |  |  |  |  |
| Protein synthesis and degradation | | | | |  |  |
| b0437 | *clpP* | 2.16 ± | 0.10 | 2.40 ± | 0.05 | ATP-dependent serine protease proteolytic subunit, part of the ClpAP, ClpAPX and ClpXP protease complexes |
| b1089 | *rpmF* | 0.01 ± | 0.02 | 0.14 ± | 0.00 | 50S ribosomal subunit protein L32 |
| b3294 | *rplQ* | > 0.01 ± | 0.00 | 0.01 ± | 0.00 | 50S ribosomal subunit protein L17 |
| b3308 | *rplE* | 1.27 ± | 0.22 | 1.16 ± | 0.22 | 50S ribosomal subunit protein L5 |
| b3311 | *rpsQ* | 1.14 ± | 0.34 | 2.03 ± | 0.39 | 30S ribosomal subunit protein S17 |
| b3317 | *rplB* | 0.88 ± | 0.16 | 0.94 ± | 0.03 | 50S ribosomal subunit protein L2 |
| b3319 | *rplD* | 1.03 ± | 0.19 | 1.06 ± | 0.02 | 50S ribosomal subunit protein L4; erythromycin sensitivity |
| b3320 | *rplC* | 0.86 ± | 0.09 | 0.92 ± | 0.07 | 50S ribosomal subunit protein L3 |
|  |  |  |  |  |  |  |

Values represent the fold-change in the expression of the gene of interest relative to the internal control (*gapA*) in the FtsZ-deprived (120 min) or the FtsZ-restored (90 min) samples compared to unperturbed cells. Data, presented as 2^-∆∆Ct^, are the average ± standard deviation from at least three independent experiments (independent cultures, RNA extractions and qRT-PCR) for each condition. The *ftsZ* transcript level was monitored as control. Stars indicate LexA-regulated promoters inferred experimentally (Fernández De Henestrosa AR *et al.* Mol Microbiol **35**:1560-1572. 2000) or based on similarity to consensus sequence (http://ecocyc.org/). Genes within each functional group are ordered accordingly to their genome position (b number; Blattner FR, *et al.* Science **277:**1453-1462. 1997). Product descriptions were taken from the EcoCyc database (Keseler IM *et al.* Nucleic Acids Res **37**:D464-4703. 2009). Vertical lines group genes belonging to the same operon.
